# Salvianolic acid B combined with bone marrow mesenchymal stem cells piggybacked on HAMA hydrogel re-transplantation improves intervertebral disc degeneration

**DOI:** 10.3389/fbioe.2022.950625

**Published:** 2022-09-27

**Authors:** Jie Hu, Cai Li, Shichang Jin, Yuchen Ye, Yuekun Fang, Panpan Xu, Changchun Zhang

**Affiliations:** ^1^ The First Affiliated Hospital of Bengbu Medical College, Bengbu, Anhui, China; ^2^ Key Laboratory of Tissue Transplantation in Anhui Province, Bengbu Medical College, Bengbu, Anhui, China; ^3^ Bengbu Medical College, Bengbu, Anhui, China

**Keywords:** BMSCs, HAMA, SALB, IDD, hydrogel

## Abstract

Cell-based tissue engineering approaches have emerged as a realistic alternative for regenerative disc tissue repair. The multidirectional differentiation potential of bone marrow mesenchymal stem cells (BMSCs) to treat disc degeneration intervertebral disc degeneration has also become a viable option. We used 1% HAMA hydrogel as a carrier and co-encapsulated BMSCs and Salvianolic acid B (SalB) into the hydrogel to reduce the apoptosis of the transplanted cells. The protective effect of SalB on BMSCs was first verified *in vitro* using the CCK8 method, flow cytometry, and Western-Blotting, and the physical properties and biocompatibility of HAMA hydrogels were verified *in vitro*. The rat model was then established using the pinprick method and taken at 4 and 8 W, to examine the extent of disc degeneration by histology and immunohistochemistry, respectively. It was found that SalB could effectively reduce the apoptosis of BMSCs *in vitro* by activating the JAK2-STAT3 pathway. 1% HAMA hydrogels had larger pore size and better water retention, and the percentage of cell survival within the hydrogels was significantly higher after the addition of SalB to the HAMA hydrogels. In the *in vivo* setting, the HAMA + SalB + BMSCs group had a more pronounced delaying effect on the progression of disc degeneration compared to the other treatment groups. The method used in this study to encapsulate protective drugs with stem cells in a hydrogel for injection into the lesion has potential research value in the field of regenerative medicine.

## 1 Introduction

Due to the increasing aging of our society, degenerative disc disease has become one of the main causes of back and leg pain in people (Andersson, 1999). The current treatment options for degenerative disc disease are surgery and conservative treatment; however, both treatments can only relieve the symptoms of disc degeneration and fail to restore the normal biological properties of the disc. With the rapid development of genetic engineering, stem cell technology, and tissue engineering technology (Lazebnik et al., 2011; Hudson et al., 2013), the use of new technologies to treat degenerative disc disease offers the possibility of restoring the normal biological function of the disc.

The intervertebral disc (IVD) is composed of a central portion of nucleus pulposus (NP) tissue, a fibrous ring structure that encases the NP, and cartilage endplates at the upper and lower ends (Gan et al., 2017). It is now believed that the onset of disc degeneration is mainly associated with the degeneration of the nucleus pulposus under mechanical stress, which causes massive apoptosis of the nucleus pulposus cells and local inflammatory response of the disc, and the degradation of the extracellular matrix (Yamada et al., 2022). Studies have shown that the percentage of apoptotic cells in degenerating discs is as high as 53%–73% (Kokubun et al., 1996); so, the most important factor in the degenerative process of the disc is the massive apoptosis of the nucleus pulposus cells (NPCs). Finding how to increase the number of locally surviving cells during degeneration is the key to treatment.

The BMSCs are a class of cells with multidirectional differentiation potential that can differentiate in different directions under different induction conditions. Related research (Ukeba et al., 2022)found that after co-culture of BMSCs with NPCs for 1 week, the expression of NPCs-specific marker genes in BMSCs increased significantly, demonstrating that the influence of the NPCs can promote the specific differentiation of BMSCs toward NPCs. At the same time, the hypoxic environment within the degenerated discs has a beneficial effect on the proliferation of BMSCs and their differentiation toward NPCs (Sinkemani et al., 2020; Samal et al., 2021). Therefore, it is highly feasible to use localized BMSCs transplantation to delay and treat intervertebral disc degeneration (IDD). However, in degenerated discs, the cellular environment is exceptionally harsh, and as degeneration progresses, nutrients are reduced and metabolites accumulate in the disc, causing abnormal changes in factors such as pH, oxygen partial pressure, and osmolality in the disc (Sun et al., 2019). Although transient mild hypoxia can promote cell proliferation, long-term severe hypoxia and inflammatory infiltration can cause massive death of the original NPCs and injected BMSCs (Wang et al., 2015); so, how to solve the survival problem of transplanted BMSCs is an important part of ensuring the repair effect.

Direct transplantation of stem cells to the lesion site is not conducive to cell survival due to the local microenvironment. With the development of tissue engineering technology, injectable light-sensitive hydrogels are gradually developed and matured. As an ideal scaffold material, besides providing mechanical support to the degenerated disc, it should have good biocompatibility, respect the morphology and function of the cells, and provide a good three-dimensional growth environment for the cells. Hyaluronic acid (HA) is a component widely found in the extracellular matrix (ECM). It is a non-immunogenic, degradable, and highly biocompatible natural linear polymer (Taz et al., 2019; Ying et al., 2019) that plays an important role in biological processes such as tissue engineering (Kenar et al., 2019), drug delivery (Sahiner et al., 2019; Yegappan et al., 2019), and immunomodulation (Yu et al., 2018; Li et al., 2019). In addition, hyaluronic acid has good water retention properties. Agarwal, G et al. (2020) found that hyaluronic acid hydrogels cured with the addition of a cross-linker retained the excellent biological properties of hyaluronic acid (HA) itself, and when used as a stem cell carrier, it can regulate the function of cells in the hydrogel and mimic the extracellular matrix, which has a positive effect on the survival of cells and the repair of cartilage and other tissues. Therefore, HAMA hydrogel can be used as a good carrier for BMSCs in the repair of IDD.

It has been shown that the encapsulation of BMSCs in hydrogel alone does not prevent the damage to transplanted cells by the local environment of the lesion (Yang et al., 2010); so, we introduced a drug that can improve the local microenvironment to provide a protective effect on the cells by co-encapsulating it with BMSCs in the hydrogel. Therefore, we introduced salvianolic acid B (SalB) as an antioxidant to protect BMSCs and reduce their apoptosis. It was shown that salvianolic acid B (molecular weight 718.62), the active ingredient in *Salvia miltiorrhiza*, protects against stem cell injury induced by oxidative stress (Lu et al., 2010; Shu et al., 2015) and contributes to the survival and self-renewal of bone marrow-derived stem cells (Bi et al., 2008; Zhang et al., 2012). Kim et al. (2018) demonstrated that salvianolic acid B significantly increased the activity of foreign transplanted BMSCs and protected them from the harsh microenvironment of the damaged area. Related studies (Wang F. et al., 2019; Gao et al., 2019; Ko et al., 2020) confirmed that Sal B can significantly inhibit autophagy and reduce apoptosis, and it also has a good inhibitory effect on oxidative stress in cells induced by hydrogen peroxide (Liu et al., 2016; Xu et al., 2016; Katary et al., 2019), which can significantly reduce the production of reactive oxygen species and increase the secretion of related oxidation-inhibiting enzymes such as SOD and GPX.

The strong oxidative environment in degenerated discs (Feng et al., 2017; Bai et al., 2020; Wang et al., 2021) is an important factor causing massive apoptosis in transplanted BMSCs, and the JAK2/STAT3 pathway is a signal transduction pathway for several cytokines (Kim et al., 2015) and is also closely related to apoptosis. However, it is still unknown whether the JAK2/STAT3 pathway is involved in the protective process of SalB against BMSCs. Therefore, in this study, the protective effect of SalB on BMSCs and its mechanism were evaluated *in vitro* using an H_2_O_2_-induced apoptosis model of bone marrow MSCs. *In vivo*, Sal B and BMSCs were co-encapsulated in HAMA hydrogel and injected into the degenerated rat caudal intervertebral discs to examine the repair effect of BMSCs on the degenerated discs under the protection of Sal B.

## 2 Materials and methods

### 2.1 Reagents

Sal B was purchased from Maclean’s Biochemistry (China), dissolved in DMSO, and stored in the refrigerator at −80°C before use, The annexin V/FITC apoptosis kit was purchased from KGI (China), fetal bovine serum was purchased from Clark Bioscience (United States), DMEM/F12 was purchased from Biosharp (China), HAMA-400K hydrogel was purchased from Suzhou Yongqinquan Intelligent Equipment Co., Ltd. (China), CD105-APC purchased from EXBIO (Czech Republic), CD34-FITC antibody was purchased from Santa Cruz Biotechnology (United States), CD45-PE was purchased from Invitrogen (United States), CD90-FITC was purchased from Biolegend. JAK2, p-JAK2, STAT3, p-STAT3, BAX, and Bcl2 antibodies were purchased from Affinity Biosciences (China), DAPI and FITC-labeled ghost cyclic peptides were purchased from Sloarbio (China), and the Calcein-AM/PI Double Stain Kit was purchased from Yeasen Biotechnology (China), Sprague-Dawley (SD) rat bone marrow MSC osteogenesis, adipogenesis, and chondrogenesis differentiation kits were purchased from Cyagen Biotech (China).

The SD rats used in this experiment were purchased from Hunan Sleek Jingda Laboratory Animal Co., Ltd. (license number SCXK (Xiang) 2019-0004) and approved by the Ethics Committee of Bengbu Medical College (approval number: Lunde Keji [2020] No. 198).

### 2.2 *In vitro*

#### 2.2.1 Cell extraction, culture, and identification

SD rat primary BMSCs were extracted from the bone marrow of 4-week-old male SD rats under aseptic conditions. The bone marrow cavity was flushed with 5 ml DMEM/F12 using a sterile 5 ml syringe inserted into one end of the bone marrow cavity, centrifuged and the precipitate was washed with a DMEM/F12 medium supplemented with 10% FBS and 1% penicillin-streptomycin, placed in a 25 cm^2^ culture flask and incubated at 37°C, 5% CO2, and 90% humidity. After 48 h, we removed the unadhered cells and replaced them with a fresh medium. The medium was changed every 3 days. When the cell fusion reached 70%–80%, the cells were rinsed with PBS; then, 1 ml of trypsin containing 0.25% EDTA was added and incubated for 1 min at 37°C. The digestion was terminated by adding 1 ml of a complete medium, and the cells were resuspended by adding 3 ml of the medium after centrifugation and passaged at 1:3. Third generation to fifth generation cells were used for the experiment. Typical surface markers expressed by bone marrow MSCs were detected via flow cytometry, using the following antibodies: CD105-APC, CD90-FITC, CD34-FITC, and CD45-PE.

### 2.3 BMSCs differentiation ability assay

The adipogenic, osteogenic and chondrogenic abilities of BMSCs were examined using Oil Red O, Alizarin Red and Alcian Blue staining before using BMSCs for *in vivo* and *in vitro* experiments, respectively. BMSCs were grown in six-well plates at a density of 2×10^4^/cm2 and cultured until complete fusion. Before performing adipogenic induction, lipogenic differentiation basal medium solutions A and B were prepared with dexamethasone, insulin, IBMX, rosiglitazone, penicillin-streptomycin, glutamine, and fetal bovine serum to form lipogenic induction differentiation medium solution A and B, respectively. First, we cultured with solution A for 3 days and then used solution B for 24 h; so, after 20–27 days of alternating culture, we fixed the cells with 4% paraformaldehyde, and stained them with oil red O.

For osteogenic differentiation, ascorbic acid, sodium β-glycerophosphate, fetal bovine serum, penicillin-streptomycin, glutamine and dexamethasone were first added to the osteogenic differentiation basal medium in proportion. After treating the bottom of 6-well plates with 0.1% gelatin, BMSCs were planted in the treated plates at a density of 2×10^4^/cm^2^, and the osteogenic differentiation complete medium was added when the cell fusion reached 60%–70%, changed every 3 days, fixed with 4% paraformaldehyde and stained with Alizarin red after 2–4 weeks of incubation.

For chondrogenic differentiation, dexamethasone, ascorbic acid, ITS additive, sodium pyruvate and proline were added proportionally to the chondrogenic differentiation basal medium to make a premix, and then TGF-β3 was added proportionately to make a complete medium for chondrogenic differentiation. After centrifugation, 3- 4×10^5^ cells were placed in 15 ml centrifuge tubes and washed twice with the premix, then resuspended with the complete medium and centrifuged; thereafter the medium was changed every 2–3 days. After 21–28 days of culture, the cells were fixed with 4% paraformaldehyde, dehydrated and embedded, and stained with Alcian blue after sectioning.

### 2.4 Cell viability determination

Plant BMSCs in 96-well plates at a density of 6,000/well, treat cells with different concentrations of H2O2 (25, 50, 100, 200, 300, 400, 500, 600, and 700 μM) for 24 h, determine the cytotoxicity of H_2_O_2_ using MTT, calculate the semi-inhibitory concentration of H_2_O_2_.

Cells were treated at a density of 4,000/well for 24, 48, and 72 h after celling with different concentrations of SalB (0.01, 0.1, 1, 10, 100 μM) with the CCK-8 method after cell planting. Cells were then treated with SalB of the above concentration gradient for 24, 48, and 72 h before using H_2_O_2_ to induce cells for 24 h. Cytotoxicity was detected using the CCK-8 method, and cell viability was calculated. The cell viability rate (%) = (OD spiked-OD blank)/(OD control-OD blank) × 100%.

### 2.5 Apoptosis detection

After cell treatment according to the control group, H_2_O_2_ group, SalB + H_2_O_2_ group, and SalB + WP1066 + H_2_O_2_ group, cells were collected, washed twice repeatedly, resuspended using Binding Buffer, incubated with Annexin V-FITC and PI for 5 min at room temperature and protected from light, and detected using flow cytometry.

### 2.6 Cell differentiation assay

BMSCs were processed according to the control group, SalB group, sham group, sham + SalB group. No treatment was performed in the control group, only SalB was added in the SalB group, rat BMSCs chondrogenic differentiation medium was added in the sham group, and in the sham + SalB group, we added SalB to it on the basis of the sham group. After 7 days of induction, staining was performed using Alcin blue.

#### 2.6.1 Western -blotting

Proteins were extracted according to the above grouping and, after quantification by BCA, the expression levels of JAK2, p-JAK2, STAT3, p-STAT3, BAX, and Bcl2 in BMSCs were detected by conventional Western blot analyses. After closure in 5% skim milk, the PVDF membranes were incubated overnight at 4°C with the following anti-rat primary antibodies: JAK2 (1:1000), pJAK2 (1:1000), STAT3 (1:1000), p-STAT3 (1:1000), BAX (1:1000), Bcl2 (1:1000), and GAPDH (1:5000), and then incubated with HRP-coupled secondary antibodies at room temperature for 2 h. Finally, the grayscale values of all bands were normalized using GAPDH.

#### 2.6.2 HAMA hydrogel physical property testing

To examine the internal morphology of the hydrogel, it was lyophilized after photocoagulation, cut off with a razor blade, and the internal morphology was examined using a scanning electron microscope.

The water retention of hydrogel was tested by weighing the weight of the hydrogel as W_0_ after it was cured in the mold, and then it was placed in a constant temperature environment and weighed as W_t_ at the 1st, 2nd, 4th, 8th, 12th, 24th, and 36th h, and then the sample was completely dried and weighed as W_a_, and the water content was calculated. The water content is calculated as: water content (%) = $\frac{Wt-Wa}{W0}*100\%$ .

For the hydrogel degradability assay, the hydrogel was cured and molded using a mold and weighed as W_0_. After the sample was placed in lysozyme and incubated at 37°C, it was weighed at the 2^nd^, 4^th^, 6^th^, 8^th^, 10th, and 12th h and recorded as W_t_, and its degradation percentage was calculated. Using the formula: degradation percentage (%) = $\frac{W0-Wt}{W0}*100\%$ .

### 2.7 Biocompatibility of HAMA hydrogel

BMSCs were mixed with 1% and 2% hydrogels at a density of 2.0×10^6^/ml and photocoagulated into shape using a mold, placed in a complete medium containing 10% fetal bovine serum, and changed every 2 days. They were stained with AM/PI live dead cells on days 1, 3, and 7 to observe intra-hydrogel cell viability. The cytoskeleton was stained on day 7 with DAPI and FITC-labeled phalloidin to observe cell morphology in different concentrations of hydrogels.

### 2.8 Cell viability assay in HAMA hydrogel

BMSCs were mixed with hydrogel at a density of 1.0 × 10^6^/ml and planted in 96-well plates at 30 μl mixture per well, photogelated and 100 μL medium was added. They were divided into HAMA and HAMA + SalB groups and cell viability rate was measured on days 1,3 and 7 using the CCK8 method respectively. Alternatively, they were divided into HAMA, HAMA + H_2_O_2_ and HAMA + SalB + H_2_O_2_ groups and cell viability rate was measured using the CCK8 method on days 1,3 and 7 respectively.

### 2.9 *In vivo*

#### 2.9.1 Establishment of the rat intervertebral disc degeneration model

Referring to the literature (Glaeser et al., 2020; Masuda et al., 2005), the degeneration model was established by needling segments 7–10 of the rat caudal spine using a 21G puncture needle and grouped into the normal group, control group, HAMA group, and HAMA + SalB group, HAMA + BMSCs group, and HAMA + SalB + BMSCs group, each containing six rats. The corresponding hydrogel mixture was injected into the intervertebral disc after needling according to the grouping. Then, photocoagulation was performed using optical fiber, and Co_7-10_was collected in week 4 and week 8 for subsequent experiments. All experimental steps were in accordance with the Animal Research Reports *in Vivo* Experiments (ARRIVE) guidelines.

#### 2.9.2 Histological testing

All collected experimental segments were fixed using 4% paraformaldehyde and then paraffin-embedded. Then, 7 μm sections were prepared from the median of the samples. HE staining and safranine O-fast green (SO-FG) staining were used for histological evaluation and histological scoring using the Han Bin scoring scale (Han et al., 2008).

#### 2.9.3 Immunohistochemistry

After dewaxing and antigen repair, the sections were incubated for 25 min using a 3% H_2_O_2_ solution and blocked for 30 min using BSA. Then, they were incubated overnight at 4°C with the anti-collagen II antibody and anti-aggrecan antibody, and incubated for 1 h at room temperature with HRP conjugated Goat Anti-Rabbit IgG (H + L). After DAB development, the nuclei were then re-stained with hematoxylin and the sections mounted with SweSuper Clean BioMount Medium. After microscopic photography, the IOD values were analyzed using ImageJ software.

### 2.10 Statistical analysis

Data are expressed as the mean ± standard deviation (SD) of at least three independent experiments. Statistical analyses were performed using GraphPad Prism 8.0 (GraphPad Software, Inc.). The one-way ANOVA, Student’s t-test, or two-way ANOVA was used to determine differences between groups. *p*-values of <0.05 were considered statistically significant.

## 3 Result

### 3.1 Characteristics of primary cultured rat BMSCs

**FIGURE 1:**
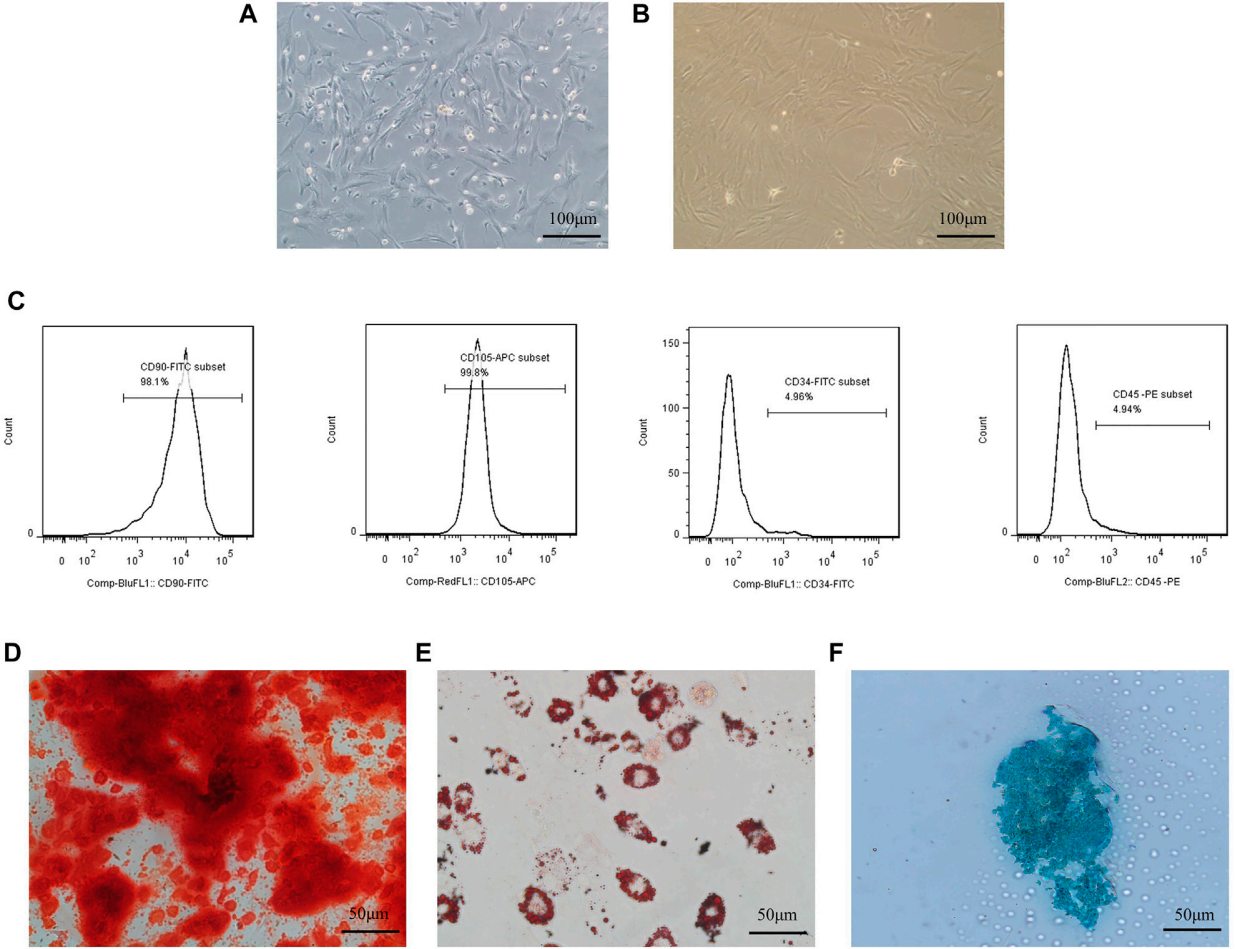
Culture and identification of BMSCs.Culture and identification of BMSCs. **(A)** Isolated primary cells showed spindle-shaped, colony-like growth; **(B)** third generation cells showed spindle-shaped appressed growth; **(C)** BMSCs surface markers were detected with high expression of CD105, CD90, and low expression of CD34 and CD45; **(D)** BMSCs stained positive for Alizarin Red after osteogenic differentiation; E:BMSCs stained positive for Alizarin Red after osteogenic differentiation; **(E)** Positive oil red O staining after adipogenic differentiation of BMSCs; **(F)** Positive Alcian blue staining after chondrogenic differentiation of BMSCs.

BMSCs extracted from SD rats are isolated by the adherent method, grown dominantly in primary cultured cells, grew spindle-like adherents, and could form fibroblast-like cell colonies (Figure 1A). On days 7–10 of the isolation culture, the cell growth and fusion degree reach approximately 80% and can be passed. Cells show a typical spindle shape (Figure 1B) when passed to the third generation; so, they are used in all experiments. To further examine the phenotype of the extracted BMSCs, multiple antigen labels on the surface of the BMSCs are analyzed using flow cytometry after labeling multiple antigens on the surface of the BMSCs. The results showed that the CD90 positivity rate reached 98.1%, the CD105 positivitye rate reached 99.8%, the CD45 positivitye rate associated with early hematopoiesis was 4.94%, and the CD34 positivity rate was 4.96% (Figure 1C).

BMSCs after osteogenesis, adipogenesis, and chondrogenesis induction after staining can be observed as deposited calcium stained red by Alizarin red dye (Figure 1D), intracellular aggregates of fat droplets stained red by Oil Red O (Figure 1E), and chondrosphere sections stained blue by Alcian blue (Figure 1F).

### 3.2 Protective effect of salvianolic acid on H_2_O_2_-induced apoptosis of BMSCs

**FIGURE 2:**
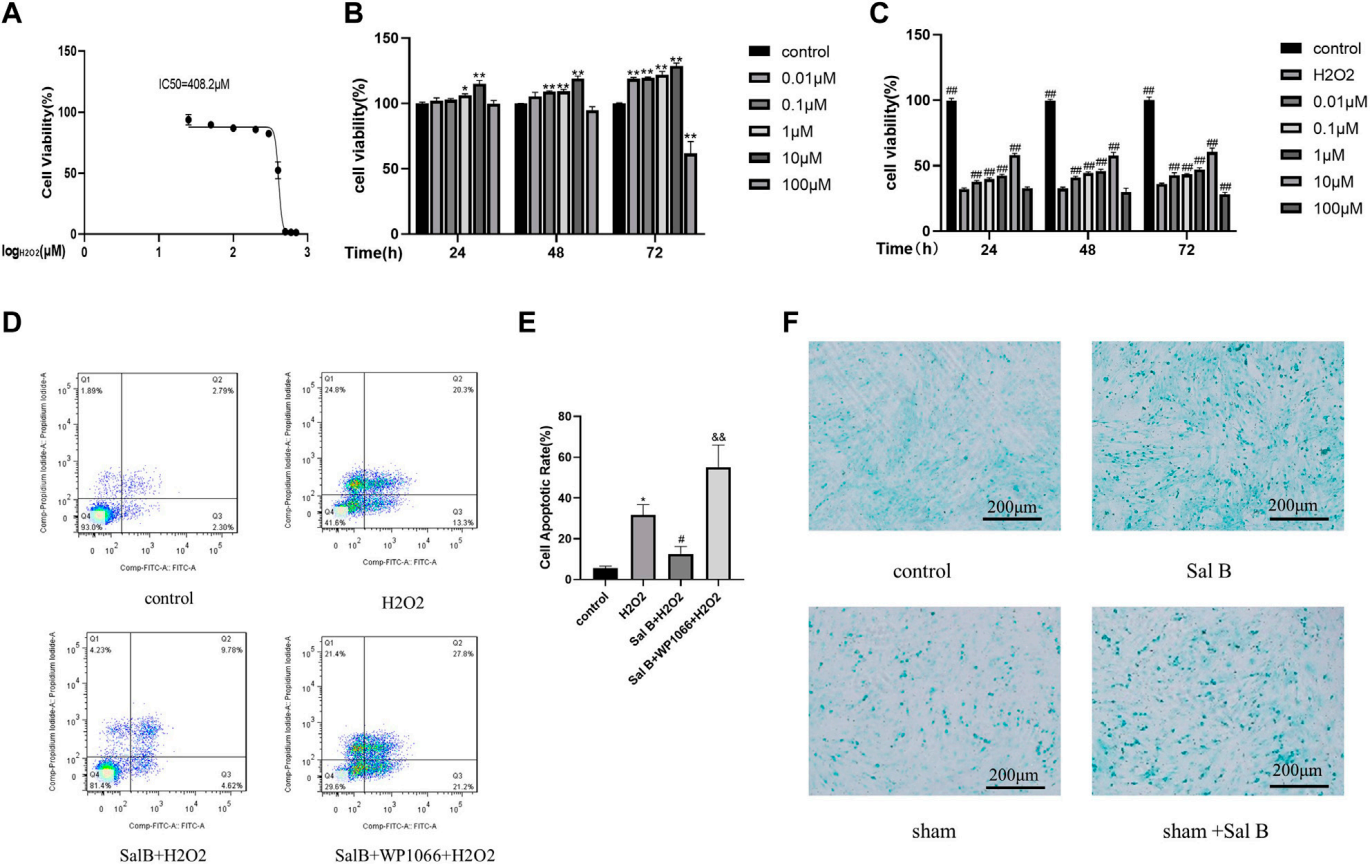
Screening of optimal action concentration of SalB.SalB optimal action concentration screening. **(A)** MTT method detects the IC50 concentration of H_2_O_2_ acting on BMSCs; **(B)** CCK8 method detects the toxic effects of different concentrations of SalB on BMSCs at 24, 48 and 72 h respectively; **(C)** CCK8 method to detect the protective effect of different concentrations of SalB on 24, 48 and 72 h for 400 μM H_2_O_2_-induced apoptosis of BMSCs; **(D–E)** flow cytometry detection showed that SalB reduced the apoptosis of H2O2-induced BMSCs. **(F)** Effect of SalB on chondrogenic differentiation of BMSCs after 7 days of induction using Alcian blue staining; *n* = 3, *: Compared with the control group, *p < 0.05, **p < 0.001; #: Compared with the H_2_O_2_ group, the #*p* < 0.05, ##*p* < 0.001; &: Compared to the Sal B + H_2_O_2_ group, &*p* < 0.05, &&*p* < 0.001.

To investigate the protective effect of SalB on the toxic effects of H2O2-induced BMSCs, we first investigated the semi-inhibitory concentration of H_2_O_2_ on BMSCs, which was derived as 408.2 μM by the MTT method (Figure 2A), and we selected 400 μM as the concentration to be used in the subsequent study.

BMSCs were treated with different concentrations of SalB (0.01, 0.1, 1, 10, and 100 μM) for 24, 48 and 72 h, respectively, and the toxic effects of Sal B on BMSCs were measured using the CCK8 method. It was found that all Sal B concentrations below 100 μM had a proliferative effect on BMSCs (Figure 2B), and the proliferative effect increased with increasing treatment time. Below 10 μM, the promotion effect of Sal B on proliferation was dose-dependent and time-dependent. However, when the concentration increased to 100 μM, the promotion effect on proliferation began to weaken. However, when the concentration was increased to 100 μM, the proliferative effect diminished, and with time, 100 μM Sal B showed some toxic effects on BMSCs, inhibiting cell proliferation.

Afterwards, we investigated the protective effect of different concentrations of Sal B on 400 μM H_2_O_2_-induced damage in BMSCs. After Sal B pretreatment and H_2_O_2_ induction, it was found that cell survival was significantly lower in the H_2_O_2_-treated group compared to the negative control group. In contrast, after pre-incubation with SalB, cell survival was significantly increased compared to the positive control group, again in a dose-dependent manner below 10 μM. At concentrations above 10 μM, the cell survival rate gradually decreased. When the concentration of Sal B reached 100 μM, it did not protect against H_2_O_2_-induced apoptosis at 72 h pre-incubation and showed some cytotoxicity. We concluded that the best concentration of SalB for H_2_O_2_-induced damage protection of BMSCs was 10 μM (Figure 2C), and this concentration was used in subsequent experiments.

### 3.3 Effect of salvianolic acid on the apoptosis of BMSCs

The results were obtained by staining the collected cells using the annexin V-FITC/PI apoptosis kit and detected by flow cytometry. Compared to the control group, the apoptosis rate increased significantly after induction with H_2_O_2_, from approximately 5% to over 30% (Figures 2D,E). The apoptosis rate of the SalB pre-incubation group decreased to less than 20% on this basis. This demonstrates that Sal B can reduce H_2_O_2_-induced apoptosis. Subsequently, to investigate whether the JAK2 pathway is involved in SalB against apoptosis, we used the JAK2 inhibitor WP1066, and induced the cells after incubation with SalB. We found that the apoptosis rate of cells treated with WP1066 increased significantly again to more than 40% compared to those induced by SalB alone, suggesting that the JAK2 pathway may be involved in the inhibition of apoptosis in BMSCs by SalB.

### 3.4 Effect of SalB on the direction of differentiation of BMSCs

After staining with Alcin Blue (Figure 2F), it was found that only a few cells in the control group were stained blue, whereas after SalB was added, the proportion of cells that stained positive increased significantly. Compared with the control group, there were more positive cells in the sham group, and after adding SalB to the sham group, the number of positive cells also increased significantly.

### 3.5 Western-Blotting

**FIGURE 3:**
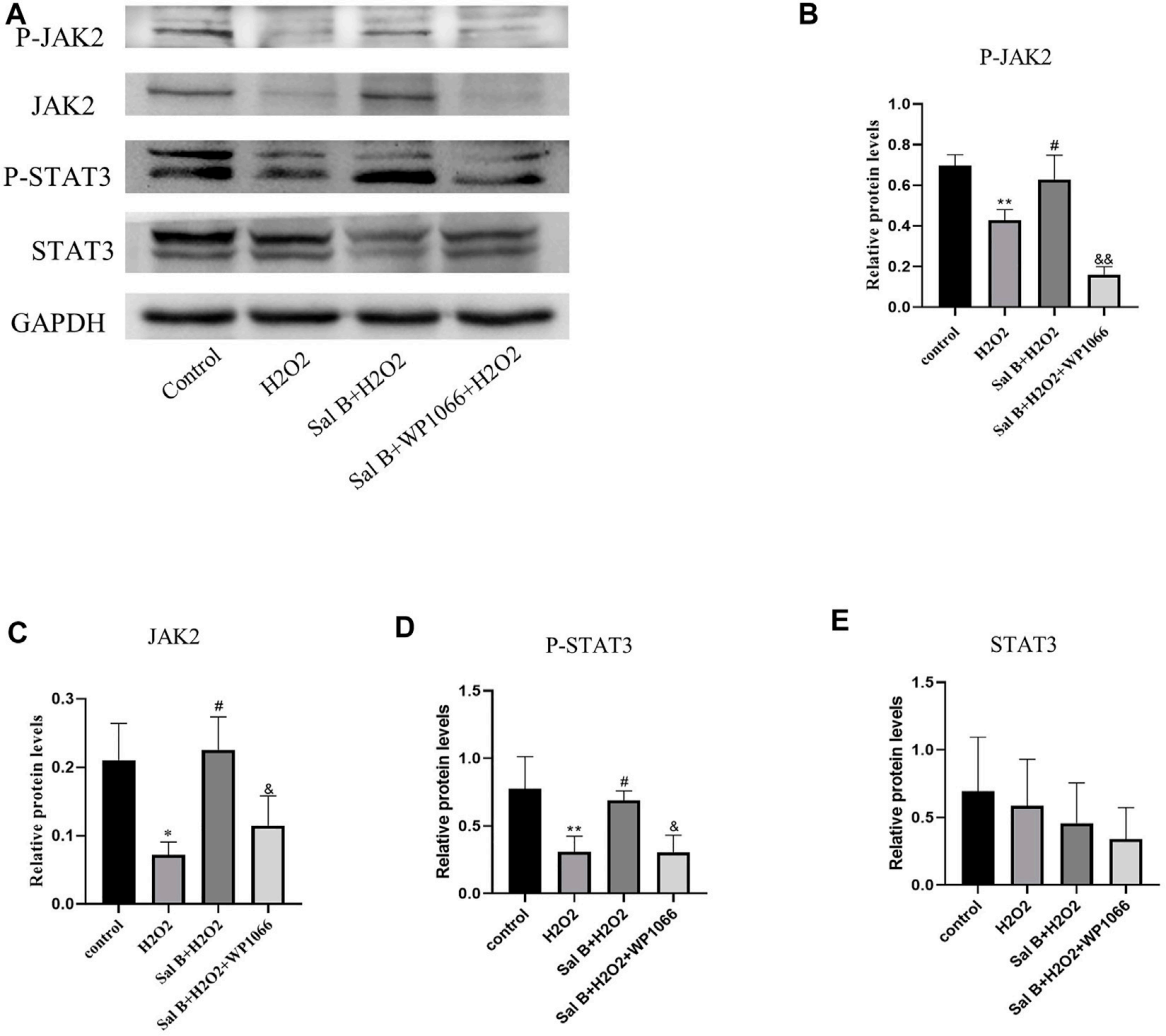
**(A)** Western-blotting assays show that SalB activates the JAK2-STAT3 signaling pathway, **(B–E)**: Western-blotting grayscale values show that SalB activates this pathway by promoting JAK2 and STAT3 phosphorylation, while WP1066 can inhibit this activation effect. *n* = 3, *: compared with control group, *p < 0.05, **p < 0.001; #: compared with H2 O 2 group, #*p* < 0.05, ##*p* < 0.001; &: compared with Sal B + H_2_O_2_ group, &*p* < 0.05, &&*p* < 0.001.

**FIGURE 4:**
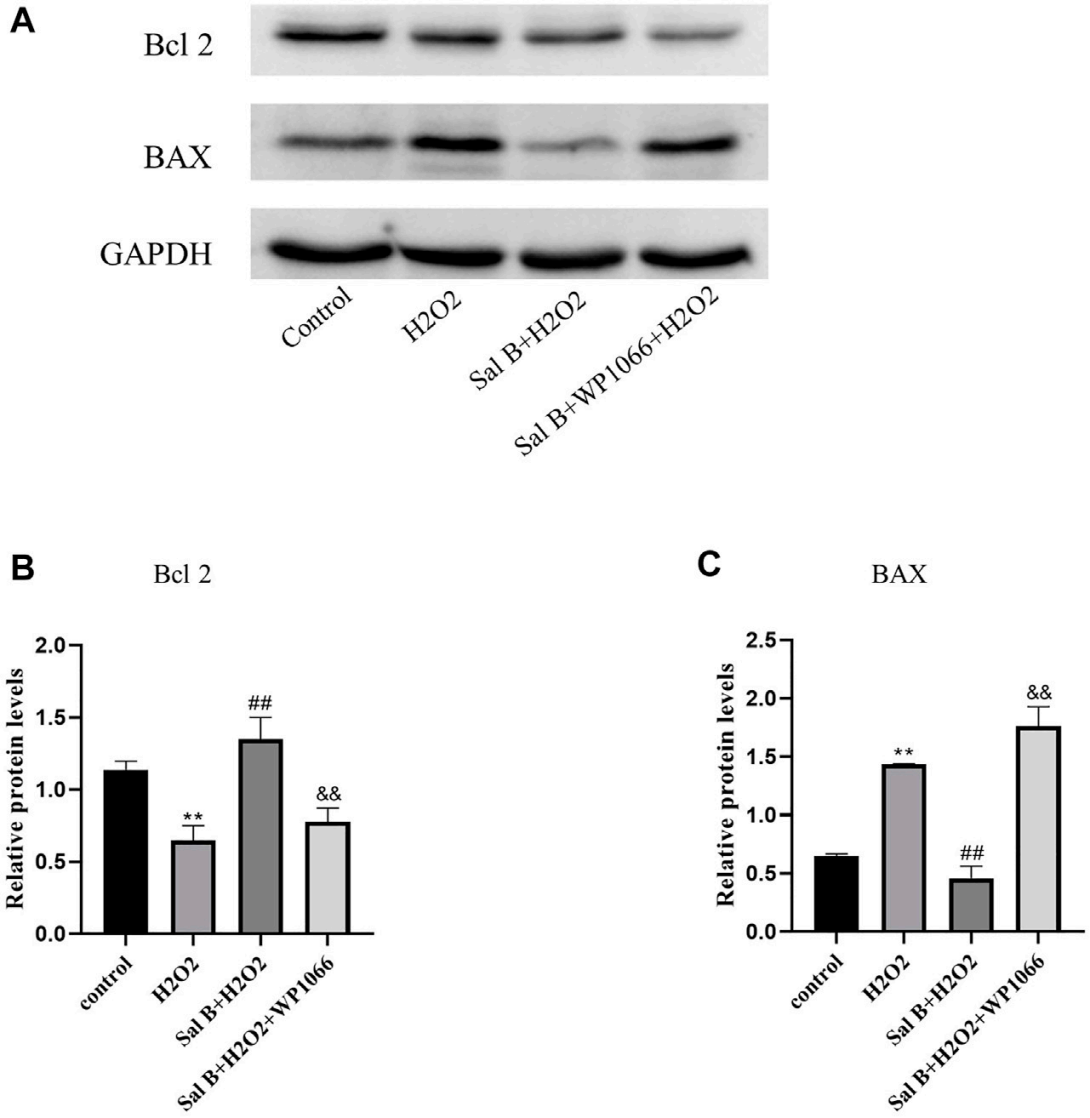
**(A)**Western-blotting detects that SalB can regulate the expression of BAX and Bcl2 proteins through JAK2-STAT3 signaling pathway, **(B,C)**: By analyzing the grayscale values of Western-blotting, it shows that SalB can reduce the expression of BAX and elevate Bcl2 at the same time, inhibit H_2_O_2_-induced apoptosis, and WP1066 could inhibit this effect .*n* = 3,*:compared with control group,*p < 0.05,**p < 0.001;#:compared with H_2_ O _2_ group, #*p* < 0.05,##*p* < &:compared with Sal B + H_2_O_2_ group, &*p* < 0.05,&& *p* < 0.001.

Western-Blotting verified the involvement of the JAK2-STAT3 pathway in the inhibition of apoptosis in BMSCs by SalB (Figure 3A). The results showed that after H_2_O_2_ stimulation, the intracellular expression of P-JAK2 (Figure 3B) and P-STAT3 (Figure 3D) was significantly reduced compared to the control group, and the expression of Bcl2 protein (Figures 4A,B) was also reduced, while the expression of BAX protein (Figure 4C) was significantly increased. This was reversed by pre-incubation with SalB prior to H_2_O_2_ induction, with increased activation of JAK2 (Figure 3C) and STAT3 (Figure 3E) proteins, resulting in a significant increase in P-JAK2 and P-STAT3 expression, an increase in Bcl2 and a decrease in BAX. This effect could be reversed by WP1066, and treatment of cells with WP1066 after preincubation with SalB again led to a further decrease in the expression of P-JAK2 and P-STAT3 and a significant increase in the amount of pro-apoptotic factors. This indicates that SalB can reduce apoptosis of BMSCs by activating the JAK2-STAT3 signaling pathway.

### 3.6 Physical characterization of HAMA hydrogels

**FIGURE 5:**
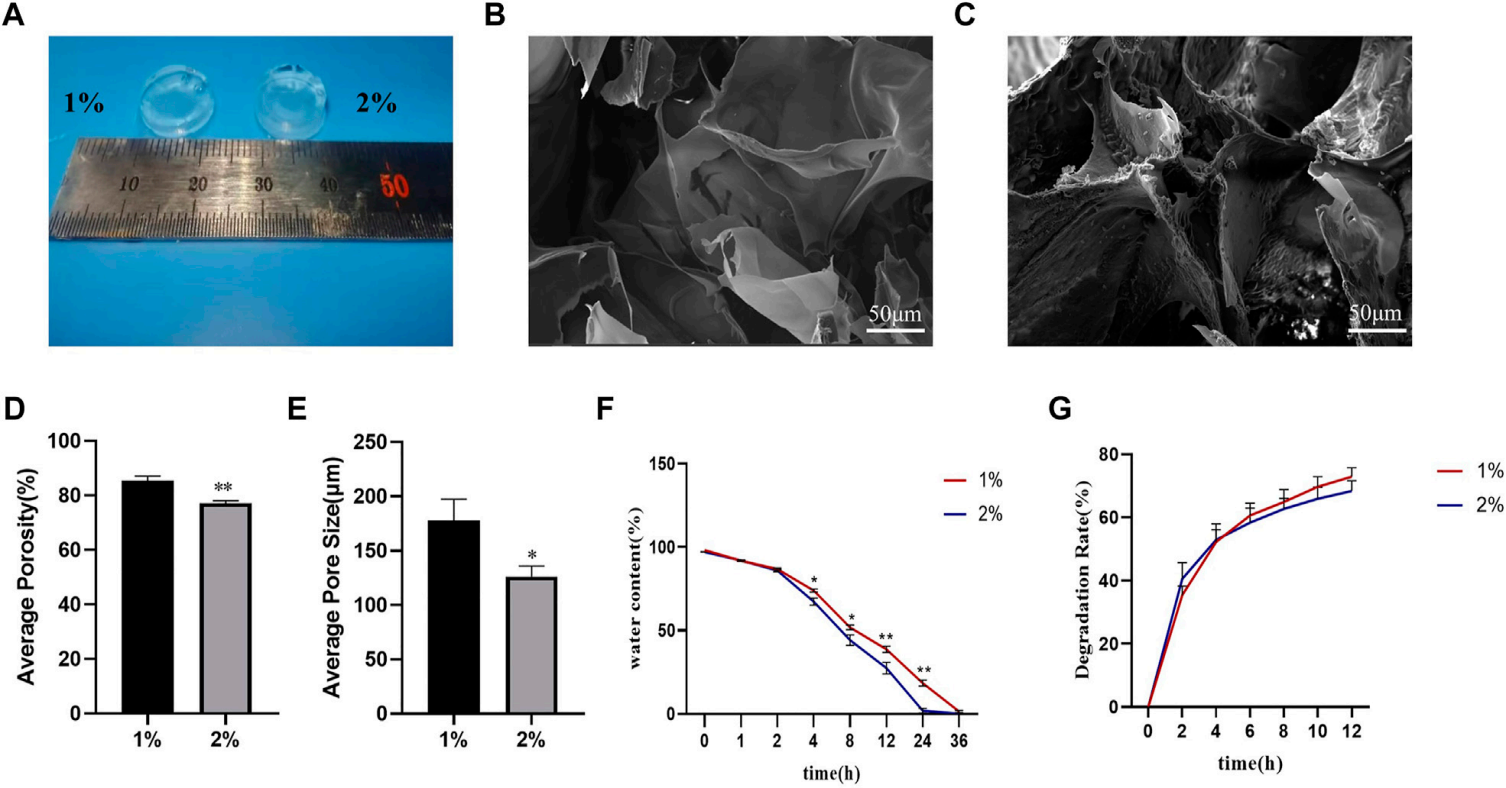
Physical properties of HAMA hydrogels with different concentrations were examined. Physical properties of HAMA hydrogels with different concentrations were examined. **(A)** Photocoagulation effect of 1% and 2% HAMA hydrogels after UV irradiation at 405 nm for 15S; **(B,C)**: Internal morphology of 1% and 2% hydrogels after photocoagulation, lyophilization, and SEM detection; **(D–E)**: Internal porosity and average pore size of 1% and 2% HAMA hydrogels; **(F–G)**: Water retention and degradation rate of 1% and 2% HAMA hydrogels were examined for water retention and degradation rate. *n* = 4, *: compared with 1%, *p < 0.05; **p < 0.001.

To verify the properties of different concentrations of hydrogels, we chose 1% and 2% HAMA hydrogels to verify their physical properties. These 1% and 2% HAMA hydrogels could be solidified and shaped by UV irradiation at 405 nm (Figure 5A). The internal morphology of the lyophilized hydrogels was scanned out using scanning electron microscopy, and both concentrations had large pore size distribution inside the hydrogels (Figures 5B,C); however, the internal pore size of 1% HAMA hydrogel was 177.89 ± 19.58 μm (Figure 5E), which was significantly larger than that of 2% hydrogel at 126.42 ± 9.58 μm. The internal porosity of the 1% hydrogel was 85.45 ± 1.66% (Figure 5D) higher than the porosity of 77.17 ± 0.98% of the 2% hydrogel.

In terms of water retention performance, we found that there was no difference in the water loss ratio between the two concentrations of hydrogels within 2 h. From the 4th hour onwards, the water loss ratio of 2% hydrogel gradually started to be higher than that of 1% hydrogel, and the gap between them gradually increased with time, and the water loss ratio of 2% hydrogel was close to 100% at the 24th hour, while at the 36th h, the 1% hydrogel was close to complete water loss (Figure 5F).

We used lysozyme to test the degradability of both concentrations of hydrogels, and the results showed that both concentrations of hydrogels could be degraded by lysozyme, and the degradation rate at 12 h was nearly 80% (Figure 5G).

### 3.7 HAMA hydrogel biocompatibility testing

**FIGURE 6:**
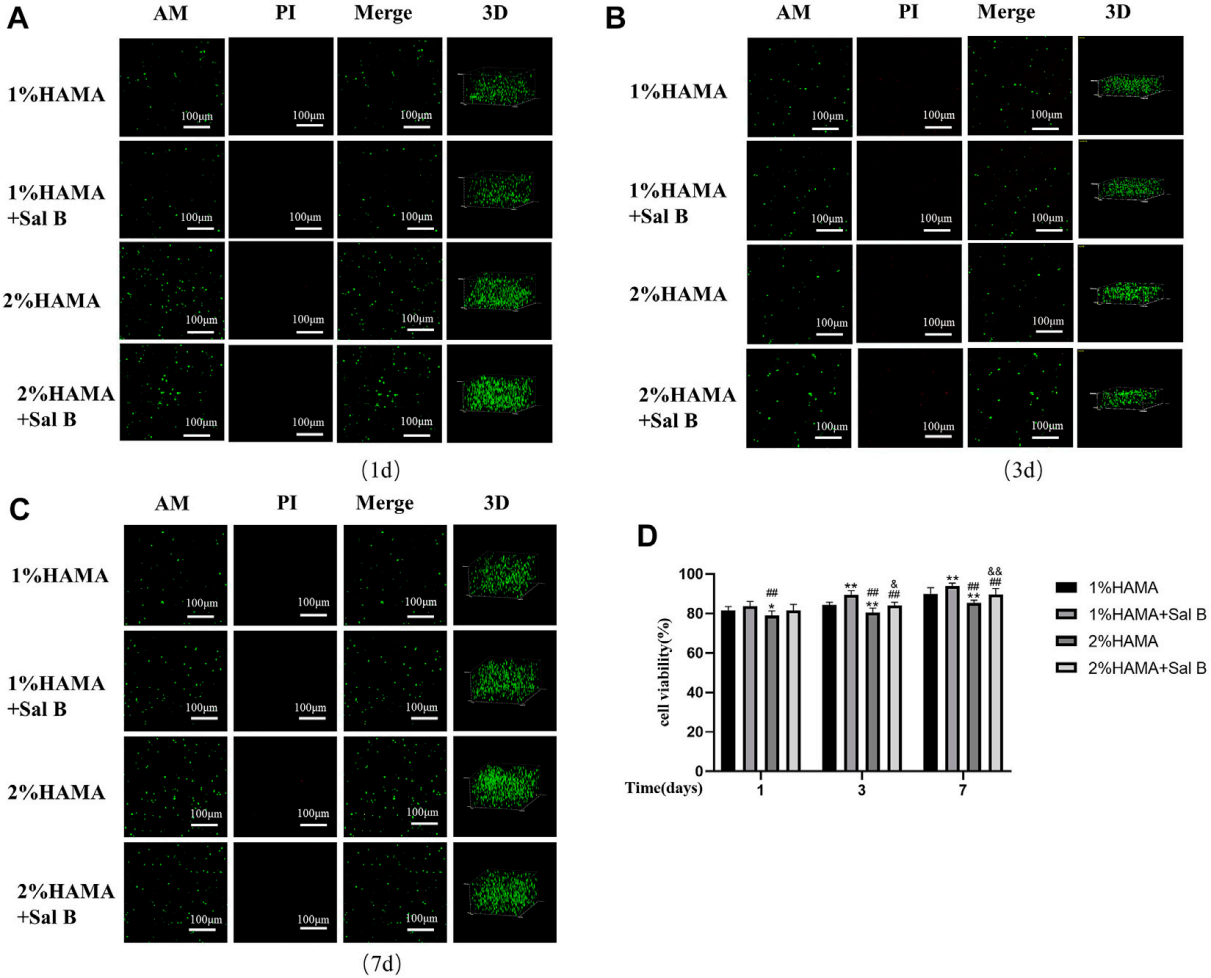
Staining of AM/PI live-dead cells in 1% and 2% HAMA hydrogels. BMSCs were mixed with 1% and 2% HAMA hydrogels, photocoagulated, cultured for 1, 3 and 7 days, labeled live and dead cells in the hydrogel using AM and PI, respectively, to measure the proportion of living cells. **(A)** Survival of BMSCs in hydrogel at 1 day of incubation; **(B)** Survival of BMSCs in hydrogel at 3 days of incubation; **(C)** Survival of BMSCs in hydrogels at 7 days of incubation; **(D)** Comparison of survival rates of BMSCs on days 1, 3 and 7 of culture; *n* = 10. *: Compared with the 1% HAMA group, *p < 0.05; **p < 0.001; #: Compared with the 1% HAMA + Sal B group, the #*p* < 0.05; ##*p* < 0.001; &: Compared with the 2% HAMA group, &*p* < 0.05; &&*p* < 0.001.

After the validation of the previous cell experiments, we concluded that 10 μM SalB has a better effect on reducing H_2_O_2_-induced apoptosis of BMSCs; so, when we wrapped BMSCs in HAMA hydrogel, we also co-wrapped this concentration of SalB in HAMA hydrogel to verify whether it could have an effect on improving the survival rate of BMSCs under the wrapping of the hydrogel (Figure 6D). At 1 day incubation, the cell survival rate in the 1% HAMA hydrogel was 81.61 ± 1.89% and increased to 83.63 ± 2.48% with the addition of Sal B (Figure 6A). The cell survival rate in 2% HAMA hydrogel was 78.97 ± 2.34% and increased to 81.47 ± 3.19% with the addition of Sal B. When the culture time was extended to 3 days (Figure 6B), the cell survival rate was 84.32 ± 1.38% in the 1% HAMA hydrogel group, 89.55 ± 2.00% in the 1% + SalB group, 80.54 ± 2.24% in the 2% HAMA hydrogel and 84.01 ± 1.67% in the 2% + SalB group. At 7 days of incubation (Figure 6C), cell survival was significantly higher compared to 1 and 3 days, with 89.98 ± 3.08% in the 1% HAMA hydrogel group, 94.04 ± 1.34% in the 1% + SalB group, 85.42 ± 1.37% in the 2% HAMA hydrogel, and 89.67 ± 2.94% in the 2% + SalB group.

**FIGURE 7:**
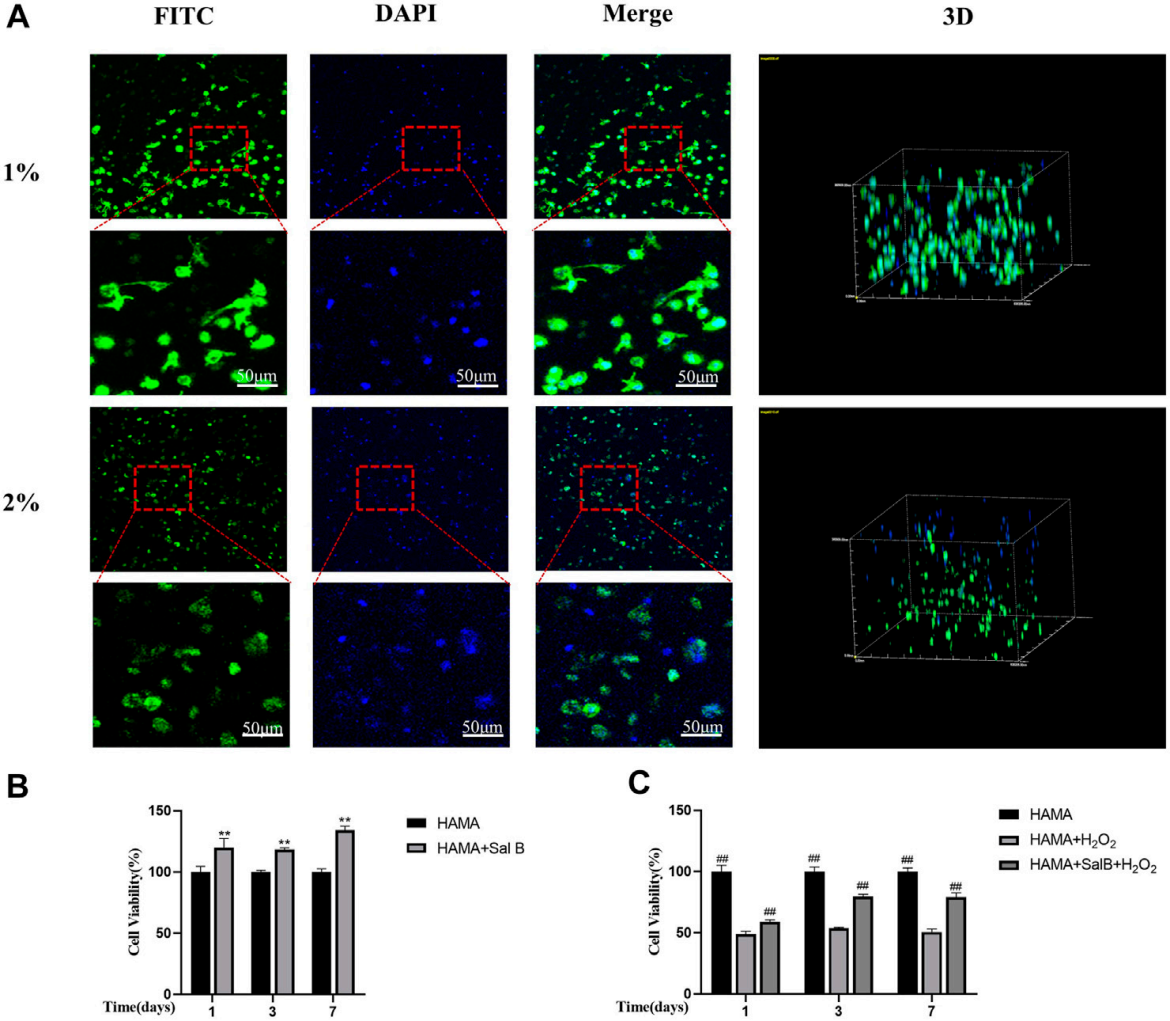
**(A)** HAMA hydrogels wrapped in BMSCs stained filamentous actin of cells using FITC-labeled phalloidin on day 7 of culture, DAPI stained the nucleus and found that in 1% HAMA some cells could be paved with cell morphology, and cells in 2% HAMA hydrogels remain spherical. *n* = 3; **(B)** CCK8 method to detect 1% HAMA hydrogel, after the addition of SalB, the proportion of cell proliferation increased with time; **(C)** The CCK8 method detects the addition of SalB in 1% HAMA hydrogels to reduce H_2_O_2_-induced apoptosis; *n* = 4, *: Compared with the HAMA group, *p < 0.05, **p < 0.001; #: Compared with the HAMA + H_2_ O _2_ group, the #*p* < 0.05, ##*p* < 0.001.

To further verify the biocompatibility of HAMA hydrogels, cells were cultured with two concentrations of hydrogels. After 7 days, microfilaments and nuclei were stained with FITC-labeled phalloidin and DAPI, respectively, and the results showed that the part of the BMSCs within the HAMA hydrogel in 1% could be slightly extended in morphology, becoming almost spindle-shaped, while BMSCs in 2% HAMA hydrogels remained spherical and did not unfold their normal cell morphology (Figure 7A).

### 3.8 Effect of SalB on cell viability within HAMA hydrogels

We measured the cell viability rate in the hydrogel by CCK8 method and found that the addition of SalB to the HAMA hydrogel significantly increased the cell viability rate compared to the HAMA group, indicating that SalB played a role in promoting cell proliferation in the hydrogel (Figure 7B). In contrast, after H_2_O_2_ treatment, the cell viability rate in the HAMA + SalB + H_2_O_2_ group was significantly higher than that in the HAMA + H_2_O_2_ group, and the cell viability rate increased gradually as the incubation time was extended (Figure 7C).

### 3.9 Histological examination

**FIGURE 8:**
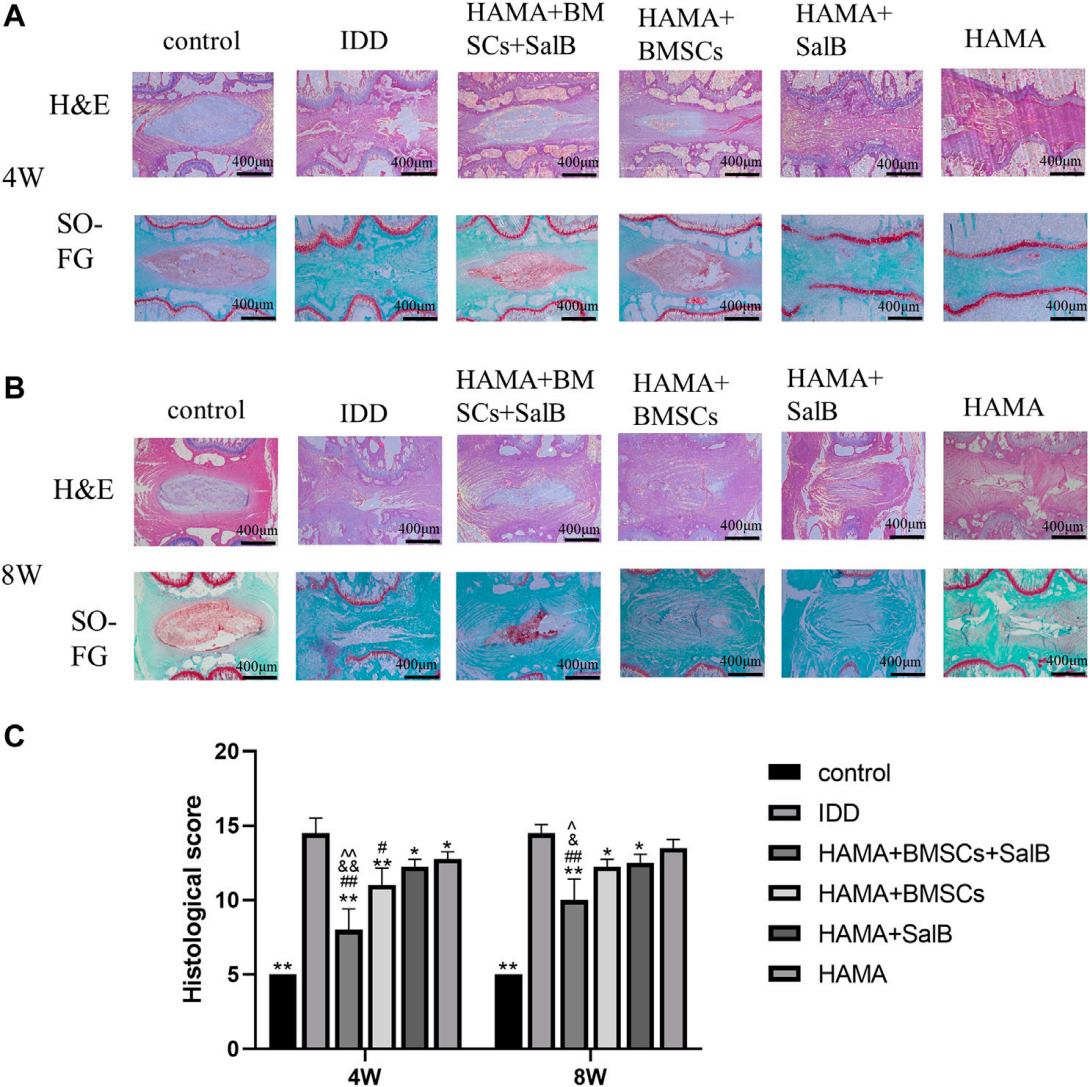
Histological examination of the disc at 4 and 8 weeks After the 4th and 8th weeks of animal modeling, HE and SO-FG staining and histological scoring of the intervertebral discs of the obtained rat tail vertebrae were performed. **(A)** HE and SO-FG staining at 4 weeks; **(B)** HE and SO-FG staining at 8 weeks. **(C)** Histological scores for 4 and 8 weeks *n* = 4, *: Compared with the IDD group, *p < 0.05; **p < 0.001; #: Compared with the HAMA group, #*p* < 0.05; ##*p* < 0.001; &: Comparison with HAMA + SalB group, &*p* < 0.05; &&*p* < 0.001; ^: Comparison with HAMA + BMSCs group, ^*p* < 0.05; ^^*p* < 0.001.

HE staining was found at 4 weeks (Figure 8A), and the control group showed normal nucleus pulposus tissue, without tissue disruption. The IDD group showed significant tissue degeneration, manifested by the loss of the nucleus pulposus and annulus fibrosus rupture disorder, and significant stenosis of the intervertebral disc height. The HAMA + BMSCs + SalB group, HAMA + BMSCs group, HAMA + SalB group, and HAMA group all showed a certain therapeutic effect. Compared with the IDD group, the degree of disc degeneration was lighter, and the height of the intervertebral disc was not obvious, of which the HAMA + BMSCs + SalB group had the best treatment effect. The degree of nucleus pulposus degeneration was lighter, and there was no obvious rupture of the fibrous ring. The results of SO-FG staining showed (Figure 8A) that the normal intervertebral disc tissue was filled with glycosaminoglycans and collagen fibers, and the fiber ring was intact. The model groups all underwent different degrees of degeneration, among which the HAMA + BMSCs + SalB group had a mild degree of degeneration. More glycosaminoglycan and collagen fibers were retained in the intervertebral disc, and the fiber rings were mildly disordered. The HAMA + BMSCs group also retained more glycosaminoglycans, and the fibrous rings were broken and partially protruded into the nucleus pulposus. The HAMA + SalB group and HAMA group had only a small amount of glycosaminoglycan and collagen fiber residues. There were no glycosaminoglycan and collagen fiber residues in the IDD group.

At 8 weeks of HE staining and SO-FG staining (Figure 8B), the HAMA + BMSCs + SalB group still showed the best therapeutic effect, with some nucleus medullary tissue as well as glycosaminoglycan and collagen fiber residues. The HAMA + BMSCs group and the HAMA + SalB group were close to complete degeneration, and the HAMA + BMSCs group could see partially extruded nucleus medullary tissue around the fiber ring, and there were still only a few glycosaminoglycan residues, and both the HAMA group and the IDD group had completely degenerated. Compared with the disc morphology at 4 weeks, the tissue degeneration of the intervertebral disc at 8 weeks was severe, the glycosaminoglycan in the nucleus pulposus was significantly reduced, and the ossification of the cartilage endplate at both ends was obvious.

Based on the results of histological scoring of the morphology of the disc in week 4 and 8, all treatment groups showed some therapeutic effect in week 4 (Figure 8C). Among them, the HAMA + BMSCs + SalB group showed the best therapeutic effect, with a score of 8.00 ± 1.41, which was significantly lower than the 14.50 ± 1.00 in the IDD group, and also lower than the 12.75 ± 0.50 in the HAMA group, 12.25 ± 0.50 in the HAMA + SalB group, and 11.00 ± 1.15 in the HAMA + BMSCs group. In week 8, the HAMA + BMSCs + SalB group score increased to 10.00 ± 1.41, which was still lower than 14.50 ± 0.58 in the IDD group, 13.50 ± 0.58 in the HAMA group, 12.50 ± 0.58 in the HAMA + SalB group, and 12.25 ± 0.50 in the HAMA + BMSCs group.

### 3.10 Immunohistochemistry

**FIGURE 9:**
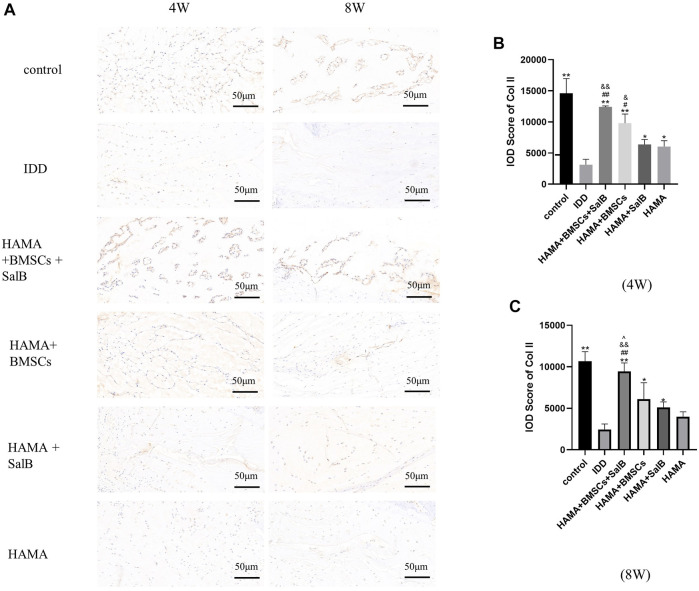
After the 4th and 8th weeks of animal model establishment, the intervertebral discs of the obtained rat tails were immunohistochemical staining, and the expression of Collagen II (Col II) in each group of intervertebral discs was detected, as well as the IOD analysis results. **(A)** The immunohistochemical staining of the discs in each group at weeks 4 and 8 was positive to varying degrees in each group. **(B)** IOD analysis results of Col II expression in each group at week 4. **(C)** IOD analysis results of Col II expression in each group at week 8. *n* = 4, *: Compared with the IDD group, *p < 0.05; **p < 0.001; #: Compared with the HAMA group, #*p* < 0.05; ##*p* < 0.001; &: Comparison with HAMA + SalB group, &*p* < 0.05; &&*p* < 0.001; ^: Comparison with HAMA + BMSCs group, ^*p* < 0.05; ^^*p* < 0.001.

Immunohistochemical staining (Figure 9A) showed that the IOD value of Col II in the HAMA + BMSCs + SalB group was 12410.00 ± 168.32 in week 4 (Figure 9B), which was significantly higher than that of the IDD group at 3133.75 ± 879.00. It was also higher than the 9800.00 ± 1481.69 in the HAMA + BMSCs group, 6,387.75 ± 782.34 in the HAMA + SalB group and 6,054.50 ± 935.73 in the HAMA group. In contrast, the IOD values for each group decreased in week 8 compared to week 4 (Figure 9C). The IOD value of the HAMA + BMSCs + SalB group was 9445.75 ± 1020.87, which was still the highest among the groups, significantly higher than the 2460.75 ± 640.27 in the IDD group, 6,115.50 ± 1965.12 in the HAMA + BMSCs group, 5122.25 ± 641.84 in the HAMA + SalB group and 3988.75 ± 596.27 in the HAMA group.

**FIGURE 10:**
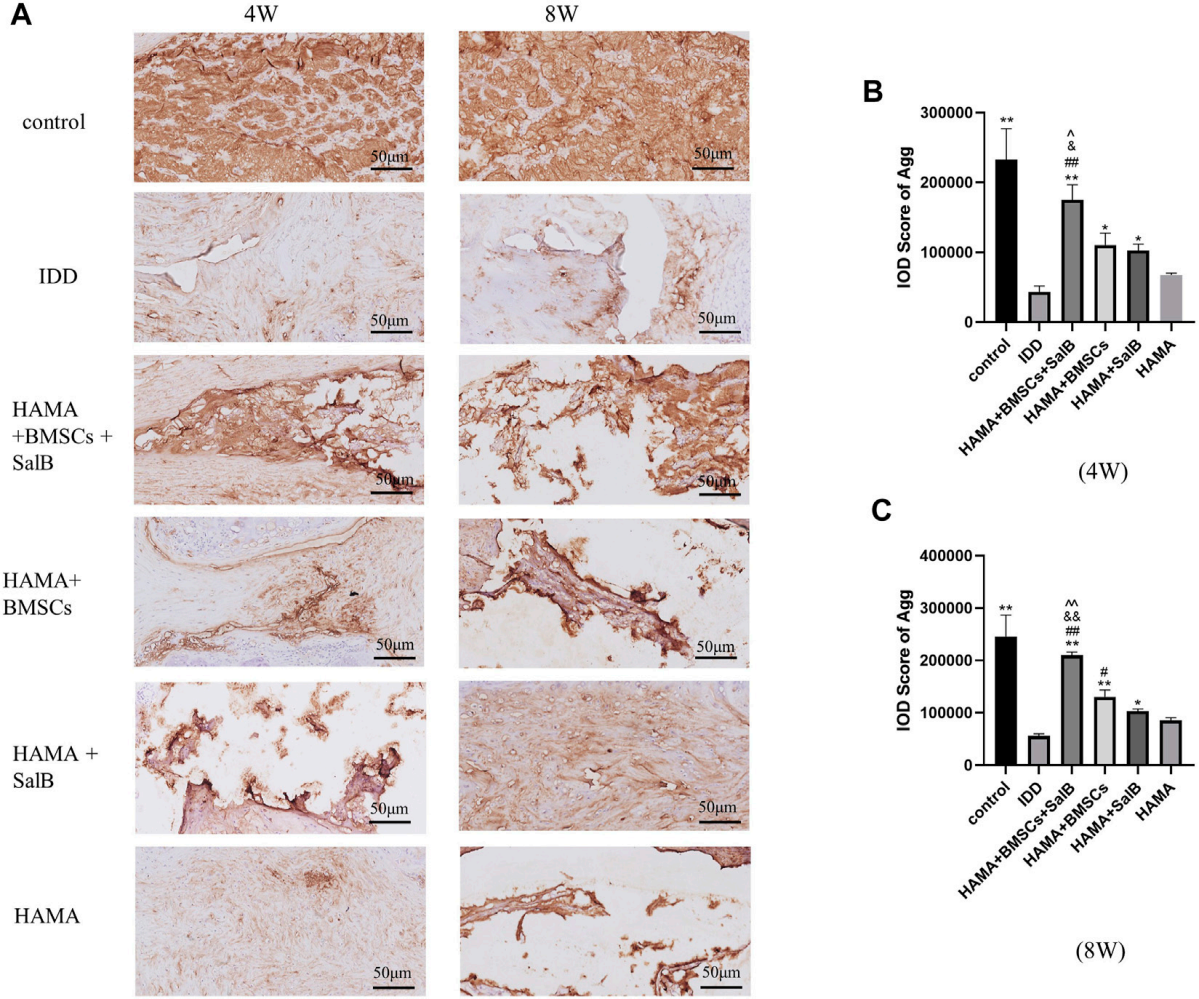
After the 4th and 8th weeks of animal modeling, the intervertebral discs of the obtained rat tails were immunohistochemical staining, and the expression of Aggrecan (Agg) in each group of intervertebral discs was detected, as well as the IOD analysis results. **(A)** The immunohistochemical staining of the discs in each group at weeks 4 and 8 was positive to varying degrees in each group. **(B)** IOD analysis results of Agg expression in each group at week 4. **(C)** IOD analysis results of Agg expression in each group at week 8. *n* = 4; *: Compared with the IDD group, *p < 0.05; **p < 0.001; #: Compared with the HAMA group, #*p* < 0.05; ##*p* < 0.001; &: Compared with HAMA + SalB group, &*p* < 0.05; &&*p* < 0.001; ^: Compared with HAMA + BMSCs group, ^*p* < 0.05; ^^*p* < 0.001.

Similarly (Figure 10A), the IOD value of Agg in the HAMA + BMSCs + SalB group was 175483.00 ± 21435.45 in week 4 (Figure 10B), which was significantly higher than that of the IDD group at 43544.75 ± 8240.11. It was also higher than the 110663.50 ± 17084.11 in the HAMA + BMSCs group, 102590.75 ± 9362.97 in the HAMA + SalB group and 67602.50 ± 2774.61 in the HAMA group. In week 8 (Figure 10C), the IOD value of the HAMA + BMSCs + SalB group was 209858.75 ± 6,247.99, which was still the highest among the groups, significantly higher than the 55739.50 ± 4313.05 in the IDD group, 130343.25 ± 13633.15 in the HAMA + BMSCs group, 103343.50 ± 4170.61 in the HAMA + SalB group and 85405.00 ± 5460.99 in the HAMA group.

## 4 Discussion

The use of cell transplantation to treat disc degeneration has long been a bone of contention. The most prominent manifestation of disc degeneration is the massive reduction in the number of nucleus pulposus cells (He et al., 2018; Hua et al., 2020), and direct replenishment of the lost NPCs is undoubtedly the most direct approach. However, some studies have shown that NPCs extracted from discs after re-transplantation lose their original ability to differentiate and synthesize type II collagen, and their application is more limited. MSCs, on the other hand, are capable of multidirectional differentiation and can be a good source of cells for disc degeneration repair (Brown et al., 2019; Clouet et al., 2019). In this study, we isolated MSCs from rat bone marrow tissues, and the isolated cells had the following characteristics: 1) the isolated cells became spindle-shaped against the wall and could form cell colonies; 2) high expression of CD105 and CD90 (>95%), and low expression of CD34 and CD45 (<5%) (Pham et al., 2018; Tian et al., 2019; Yan et al., 2021). It was also demonstrated that MSCs of bone marrow origin could differentiate toward cartilage *via* differentiation ability verification, and a previous study also demonstrated that MSCs could differentiate toward myeloid cells under the influence of a myeloid conditioned media environment, which could be applied to the repair of degenerated discs (Sinkemani et al., 2020; Felka et al., 2009). In addition to this, BMSCs can attenuate the inflammatory response of NPCs. The expression of inflammation-related factors such as IL-1 and TNF-α as well as enzymes such as MMP-13 and ADAMTS-5, that induce extracellular matrix degradation, can be found to be reduced in the co-culture system of BMSCs and NPCs compared to the culture of NPCs alone (Cao et al., 2015; Yang et al., 2015; Ouyang et al., 2017; Xia et al., 2019). Meanwhile, BMSCs and NPCs can interact with each other, and while NPCs promote the differentiation of BMSCs toward NPCs, BMSCs are able to promote the survival of residual NPCs in degenerated discs and restore their type II collagen secretion function (Vadala et al., 2008).

However, the strong oxidizing environment (Potier et al., 2007) within the degenerated discs is also a great challenge for transplanted BMSCs, and BMSCs transplantations with protective drugs or growth factors is certainly a feasible solution. SalB selected in this experiment was shown to have a good anti-apoptotic ability (Wang Q. Q. et al., 2019; Ko et al., 2020) and the ability to promote stem cell differentiation (Chen et al., 2018; Shu et al., 2018) in previous experiments. Zhao Yuan et al. found that SalB could well inhibit the MMP + -induced apoptosis of neurons and facilitated the recovery of mitochondrial function (Zhao et al., 2021). One study applied SalB directly to myeloid cells (Dai et al., 2021) and found that SalB reduced the apoptosis of myeloid cells and significantly slowed down the process of disc degeneration. Yan et al. (2020) found that using SalB co-injected with BMSCs into the degenerated disc increased the water content of the IVD and the degree of disc degeneration was reduced. At the same time, SalB can promote the differentiation of BMSCs into NPCs in the environment of the intervertebral disc. Similarly, in this experiment, we also demonstrated that SalB can promote differentiation of BMSCs into chondrocytes by adding SalB to BMSCs. However, their study only demonstrated the effect of SalB on degenerated discs from a macroscopic point of view. The effects and mechanisms of action at the tissue and protein levels were not mentioned.

In the present study we used H_2_O_2_ to simulate the strong oxidative environment in the degenerating discs and demonstrated that the use of SalB significantly reduced the apoptotic rate of BMSCs, a process in which the JAK2-STAT3 pathway is involved, and that SalB activation of JAK2 and STAT3 proteins phosphorylated directly regulated downstream apoptotic proteins. Ultimately at the cellular level using flow cytometry, a decrease in the percentage of apoptosis was observed, which explains the possible mechanism of SalB combined with BMSCs for delaying disc degeneration.

Although Yan et al. (2020) demonstrated the feasibility of SalB combined with BMSCs for the treatment of disc degeneration, a series of problems such as leakage and bone formation and insignificant therapeutic effects still need to be overcome during the establishment of animal models (Wang F. et al., 2019). In many previous studies, BMSCs or drugs are encapsulated in a carrier material, which can solve the problem of leakage when implanted into the degenerated disc, and provide a three-dimensional culture environment for the cells and a certain amount of mechanical support for the degenerated disc.

In this experiment, we used the HAMA hydrogel as a carrier to co-encapsulate SalB and BMSCs in the hydrogel, which was injected and then photocoagulated to effectively prevent the problem of leakage and provide a suitable environment for the cells to survive. There is relevant clinical evidence that after the co-transplantation of hyaluronic acid, the main component of the HAMA hydrogel, and ADSCs into patients’ degenerated discs, approximately 60% of patients showed significant improvement in their clinical symptoms within 1 year after surgery (Kumar et al., 2017). In recent years, Wang Feng et al. (Wang Q. Q. et al., 2019) injected MSCs encapsulated in hydrogel into degenerated rat intervertebral discs and observed good therapeutic effects. In this study, by examining the physical properties and biocompatibility of 1% and 2% HAMA hydrogels, it was concluded that both concentrations have larger internal pore size and porosity, which can provide a larger living space for cells, and both have better water retention and degradability, with 1% having the better performance, and both concentrations have good biocompatibility. However, wrapped BMSCs had a few cells capable of spreading inside 1% HAMA and had a higher cell survival rate. This may be due to the higher concentration of hydrogels having a stiffer matrix and thicker pore walls, which prevent the cells from spreading properly. While SalB was co-wrapped with BMSCs in HAMA hydrogels for 1, 3 and 7 days, it could be found that the cell survival rate increased significantly after the addition of SalB, whether in 1% or 2% HAMA hydrogels, and the cell survival rate in the hydrogels gradually increased with the extension of the culture time, which might be related to the proliferation of cells in the hydrogels.

Moreover, it has been demonstrated in several previous studies that the use of HAMA hydrogel piggybacking chondrocytes for cartilage repair promotes phenotypic retention and matrix synthesis of chondrocytes (Deng et al., 2020). It has also been shown that the hyaluronic acid component of HAMA hydrogel can promote chondrogenic differentiation and exostotic matrix deposition by MSCs (Amann et al., 2017). All these pieces of evidence suggest that HAMA hydrogel can be a good carrier for BMSCs and is optimal at 1% concentration. Mixing SalB with HAMA hydrogel can even significantly enhance its biocompatibility with BMSCs and promote the survival of BMSCs in the hydrogel.

The subsequent animal experiments also showed that the HAMA + SalB + BMSCs combination showed a better therapeutic effect than HAMA hydrogel wrapped with SalB or BMSCs alone. The therapeutic effect was more significant, the rate of disc degeneration in rats was significantly reduced, the retention of nucleus pulposus tissue was more complete, and the height of the disc could still be better maintained. Moreover, from the point of view of histological scores, the HAMA + BMSCs + SalB group had the lowest scores, which also proved to have the best restorative effect. At the same time, it can be found in the experiment that, influenced by the pressure in the disc, some of the nucleus pulposus tissues in the model group were gradually extruded from the fibrous ring after the animal model was established and distributed around the fibrous ring, and some of the reticular fibers could still be seen after HE staining, and some of the glycosaminoglycan residues were visible after SO-FG staining. The immunohistochemical results of Col II and Agg also showed that the HAMA + BMSCs + SalB group still retained the most Col II and Agg in weeks 4 and 8. All these pieces of evidence showed that the co-encapsulation of SalB with BMSCs in HAMA hydrogel for transplantation could show better therapeutic effects.

The present study still has a number of limitations. First, the animal model used in this experiment was the SD rat, which is still quite different from humans; thus, the results may be more representative if they are replaced by bigger mammals. Secondly, the period chosen for the animal studies was small and did not fully reflect the effect on the repair of the degenerated disc. Future experimental protocols should select multiple time points for dynamic observation to continuously track changes in disc degeneration under each treatment factor, and more studies should be conducted to investigate the mechanisms associated with *in vivo* repair in animals. Thirdly, fluorescent markers should be added to the transplanted BMSCs to track the survival of the cells.

## Data Availability

The raw data supporting the conclusion of this article will be made available by the authors, without undue reservation.

## Abbreviations

Sal B, salvianolic acid B; CCK8, cell counting kit-8; NPCs, nucleus pulposus cells; HA, hyaluronic acid, ECM, extracellular matrix; JAK2, janus Kinase 2, STAT3, signal transducer and activator of transcription 3; EDTA, ethylene diamine tetraacetic acid; IDD, intervertebral disc degeneration; BMSCs, bone marrow mesenchymal stem cells.
